# Modeling the Structure and Dynamics of Semantic Processing

**DOI:** 10.1111/cogs.12690

**Published:** 2018-10-07

**Authors:** Armand S. Rotaru, Gabriella Vigliocco, Stefan L. Frank

**Affiliations:** ^1^ Division of Psychology and Language Sciences University College London; ^2^ Centre for Language Studies Radboud University

**Keywords:** Computational modeling, Distributional textual models, Neural networks, Probabilistic models, Semantic network structure/dynamics, Lexical/semantic decision, Concreteness/imageability rating, Similarity/relatedness rating

## Abstract

The contents and structure of semantic memory have been the focus of much recent research, with major advances in the development of distributional models, which use word co‐occurrence information as a window into the semantics of language. In parallel, connectionist modeling has extended our knowledge of the processes engaged in semantic activation. However, these two lines of investigation have rarely been brought together. Here, we describe a processing model based on distributional semantics in which activation spreads throughout a semantic network, as dictated by the patterns of semantic similarity between words. We show that the activation profile of the network, measured at various time points, can successfully account for response times in lexical and semantic decision tasks, as well as for subjective concreteness and imageability ratings. We also show that the dynamics of the network is predictive of performance in relational semantic tasks, such as similarity/relatedness rating. Our results indicate that bringing together distributional semantic networks and spreading of activation provides a good fit to both automatic lexical processing (as indexed by lexical and semantic decisions) as well as more deliberate processing (as indexed by ratings), above and beyond what has been reported for previous models that take into account only similarity resulting from network structure.

## 1 Introduction

In the last two decades, important advances in our understanding of semantic memory have been achieved by the development of computational models based on the “distributional hypothesis,” introduced by Harris (1954), which claims that “words that occur in similar contexts tend to have similar meanings” (Turney & Pantel, 2010, pp. 142–143). According to this hypothesis, the collection of linguistic contexts in which a particular word occurs reflects important aspects of that word's meaning, such that commonalities in meaning between two words can be identified and even quantified by evaluating the overlap between the distributions of contexts associated with each word. For instance, the words “cat” and “dog” both frequently appear in linguistic contexts containing the words “animal”, “pet”, “furry”, “house”, and “vet”, which suggests that they are similar in meaning; in contrast, the words “vacation” and “longbow” are usually encountered in very different linguistic contexts, which makes it likely that they are semantically dissimilar. Since the distributional hypothesis does not define context in a precise manner, certain models (e.g., Topic; Griffiths, Steyvers, & Tenenbaum, 2007; LSA; Landauer & Dumais, 1997) assume that the context consists of the documents in which a given word occurs, whereas other models (e.g., HAL; Lund & Burgess, 1996; Skip‐gram, CBOW; Mikolov, Chen, Corrado, & Dean, 2013; GloVe; Pennington, Socher, & Manning, 2014) consider the words immediately following or preceding a given word to make up the context for that word.

Within the area of linguistic models, a number of studies have attempted a systematic exploration of how to best extract semantic information from linguistic contexts, by optimizing the various parameters that influence the underlying semantic model (Bullinaria & Levy, 2007, 2012; Riordan & Jones, 2011), such as the size of the linguistic corpus, the dimensionality of the semantic representations, the relative importance of each dimension, and the measure of semantic distance. Other studies have explored the benefits of including information about word order (Andrews & Vigliocco, 2010; Jones & Mewhort, 2007), syntactic dependencies (Padó & Lapata, 2007), and types of semantic relations (e.g., hypernymy; Baroni, Murphy, Barbu, & Poesio, 2010). More recently, studies have begun examining the differences between “count” models, where the vector representations reflect the linguistic contexts in which a given word appears, and “predict” models, in which the representations are designed to predict the contexts in which a given word occurs (Baroni, Dinu, & Kruszewski, 2014; Mandera, Keuleers, & Brysbaert, 2017).

Distributional models lend themselves to the investigation of patterns of semantic relations that link the individual representations within semantic memory, following original ideas by Collins and Loftus (1975) according to whom semantic memory can be regarded as a network. As a result, network analyses of semantic networks have attracted an increasing amount of attention in recent years (for reviews on linguistic/psycholinguistic applications of network science, see Borge‐Holthoefer & Arenas, 2010; Solé, Corominas‐Murtra, Valverde, & Steels, 2010; for a general review of network‐based analyses of cognition, see Baronchelli, Ferrer i Cancho, Pastor‐Satorras, Chater, & Christiansen, 2013). A number of studies have used network‐based measures such as number of neighbors or clustering coefficient to investigate (dis)similarities across domains of knowledge, such as between abstract and concrete words (Hoffman, Lambon Ralph, & Rogers, 2013; Jones, Johns, & Recchia, 2012; McDonald & Shillcock, 2001). Other studies sought to explain differences in RTs and error rates in tasks such as lexical or semantic decisions among words (Danguecan & Buchanan, 2016; Hargreaves & Pexman, 2014; Moffat, Siakaluk, Sidhu, & Pexman, 2015; Newcombe, Campbell, Siakaluk, & Pexman, 2012; Recchia & Jones, 2012). Many of these studies take advantage of large datasets of behavioral responses which are now available for English, for tasks including, but not limited to, lexical decision (Balota et al., 2007; Keuleers, Lacey, Rastle, & Brysbaert, 2012), semantic decision (Pexman, Heard, Lloyd, & Yap, 2017), concreteness ratings (Brysbaert, Warriner, & Kuperman, 2014), imageability ratings (Gilhooly & Logie, 1980; Stadthagen‐Gonzalez & Davis, 2006), as well as similarity/relatedness ratings (Bruni, Tran, & Baroni, 2014; Gerz, Vulić, Hill, Reichart, & Korhonen, 2016).

While it is encouraging to see that network‐based measures derived from distributional models can capture variance in these tasks above and beyond that explained by other lexical variables, it is also the case that they are clearly limited, as they do not take into account the dynamics of activation. The dynamics of activation are, instead, central to connectionist models developed in cognitive science to capture a variety of aspects of semantic processing (e.g., McClelland et al., 2010; Zorzi, Testolin, & Stoianov, 2013). However, most of these models do not include realistic representations but, rather, simplified ones, for computational feasibility.

Our work aims to bring together distributional models of semantic structure and processing models of lexical activation. Firstly, we model both the structural properties of semantic networks, as well as their dynamic aspects, by considering the flow of semantic activation (Anderson, 1983; Collins & Loftus, 1975) generated by the automatic processing of individual words. An important consequence of looking at both structure and dynamics is that it allows us to assess the effects of direct, as well as indirect, mediated semantic relations between words, rather than limiting our analysis to strong, direct semantic links. Previous research using models of semantics based on free association (De Deyne, Navarro, & Storms, 2013; Steyvers, Shiffrin, & Nelson, 2004) shows that indirect associations provide a complementary source of semantic information, in tasks including lexical decision, semantic similarity rating, and extralist cued recall. However, to the best of our knowledge, there are very few studies that investigate the explanatory power of indirect semantic relations in text‐based models of semantics, as well as their temporal dynamics (for an exception, see De Deyne, Verheyen, & Storms, 2016). Starting from standard distributional models of semantics, we allow activation to spread throughout the semantic network, as dictated by the patterns of semantic similarity between words, and record the activation of each word, as a function of time. We then study how the activation pattern at each time point relates to task performance in a number of tasks, as a means of linking dynamics to observable task behavior.

Secondly, we assume that both strong and weak semantic relations between words, as indexed by standard measures of semantic similarity (e.g., vector cosine), contribute to performance in semantic tasks (Chen & Mirman, 2012; Mirman & Magnuson, 2008), rather than focusing only on the strong relations, as is traditionally done when performing network analyses (Buchanan, Westbury, & Burgess, 2001; Griffiths, Steyvers, & Tenenbaum, 2007; Gruenenfelder, Recchia, Rubin, & Jones, 2016; Utsumi, 2015). The significant influence of distant neighbors is likely to be a direct result of the fact that words have considerably more distant neighbors than close ones, given that semantic similarity based on the cosine measure follows a power law distribution (Griffiths, Steyvers, & Tenenbaum, 2007). Therefore, we keep both classes of neighbors in our models, and we do not make any a priori assumptions about any privileged role that close neighbors might have over distant ones (or vice‐versa), in the course of semantic processing.

Within our dynamic models, semantic activation flows from an initial concept to its neighbors, then to the neighbors of its neighbors, and so on, until the system reaches a global “attractor” state. However, unlike many other connectionist models (Chen & Mirman, 2012; Hoffman & Woollams, 2015; Rogers & McClelland, 2004), they have a large number of nodes and feedforward/feedback/recurrent connections, making them more realistic models of human lexico‐semantic knowledge. As a result, it is expected they should provide better insight into the distinct contribution of structural and task‐related aspects of semantic behavior. Our models can also be seen as probabilistic, such that at each step, they make use of their underlying discrete‐time Markov chain, in order to perform multi‐step inferences. Thus, our approach lies at the intersection of connectionist (McClelland et al., 2010) and probabilistic (Griffiths, Chater, Kemp, Perfors, & Tenenbaum, 2010) modeling.

## 2 Model development

### 2.1 Distributional semantics models

Previous studies have shown that “word‐as‐context” models (e.g., HAL, Skip‐gram, CBOW, GloVe), provide a better fit to behavioral data, as compared to “document‐as‐context” models (e.g., LSA, Topic), in tasks such as semantic similarity rating (Bruni, Boleda, Baroni, & Tran, 2012), and semantic categorization (Riordan & Jones, 2011). In addition, a number of recent studies (Baroni et al., 2014; Pereira, Gershman, Ritter, & Botvinick, 2016) found that, within the class of “word‐as‐context” models, the CBOW and GloVe models have a clear advantage over their competitors, in tasks such as semantic similarity rating, semantic categorization, synonym detection, and analogy completion. Given that these models have shown their superiority in a number of tasks, we adopt them as our models of choice. We include both CBOW and GloVe to test whether our findings generalize beyond a specific architecture. Moreover, to further assess if our results truly support a role for the dynamics of semantic activation beyond the structural assumptions, we also include the LSA model in our analyses. For our computational experiments, we use the *gensim* tool (Řehůřek & Sojka, 2010), for the CBOW and LSA models, and the GloVe implementation provided by the authors of the model (available for download at https://github.com/stanfordnlp/GloVe).

We derive our semantic representations by training the models on the written part of the British National Corpus (BNC; Leech, Garside, & Bryant, 1994), containing approximately 87 million words. The BNC consists of contemporary texts from a variety of sources (e.g., newspapers, journals, books, letters, essays), providing a comprehensive corpus of modern British English. In order to improve the quality of the resulting representations, we first pre‐process the corpus by converting all the words to lowercase, eliminating punctuation marks and removing words whose absolute frequencies are less than five. We then construct 300‐dimensional vector representations for the words in our corpus. For reasons of computational efficiency, we do not employ all the words covered by our models, but instead keep only the 28,592 words that are also part of the 30,000 most frequent nouns, verbs, and adjectives, according the SUBTLEX‐UK frequency norms for British English (Van Heuven, Mandera, Keuleers, & Brysbaert, 2014).

### 2.2 Structure and dynamics

Since we are interested in obtaining semantic networks that reflect the semantic associations between words, we compute a representational similarity matrix *SM* (i.e., the structural model) from the vectors produced by each of the three distributional models, using vector cosine as a measure of similarity between the word representations. For each model, the matrix *SM* contains the structure of our semantic network, such that any value *SM*(*i,j*) can be interpreted as the strength of the semantic association between words *w*_*i*_ and *w*_*j*_. Within *SM*, large values (i.e., close to 1) indicate pairs of words that are close semantic neighbors, whereas small values (i.e., close to 0) correspond to pairs of words that are only weakly related. Given that negative cosine values are likely to provide very little or no useful semantic information, word pairs with negative cosine similarity receive a zero value in *SM*, as a means of reducing the amount of noise present.

The matrices *SM* represent our structural models. In order to obtain our dynamic models, we assume that semantic activation spreads throughout the networks, such that the activation propagated from the source word *w*_*i*_ to the target word *w*_*j*_ is proportional to both the current activation level of *w*_*i*_, and the value of *SM*(*i,j*), following the principle that the more similar two words are, the more activation flows between them. We also impose that the total amount of activation present in the networks should remain constant. Thus, we set to zero all the diagonal elements (we deal with these recurrent connections separately; see below) and normalize the rows of the resulting matrices *SM*_*NORM*_, such that each row sums to one (i.e., each row can be seen as estimating the conditional probability distribution over the semantic neighbors of the word associated with that row), meaning that the total activation provided by *w*_*i*_ to its semantic neighbors is exactly equal to its current level of activation. However, since it is very plausible that the source word *w*_*i*_ also retains some of its activation, we employ the weighted average of *SM*_*NORM*_, which indexes feedforward/feedback connections, and the identity matrix, with indexes recurrent connections, rather than *SM*_*NORM*_ itself. The weight (i.e., 2/3 for *SM*_*NORM*_ and 1/3 for the identity matrix) is chosen heuristically (see the study by De Deyne et al., 2016, for a similar approach). This is done in order to strike a balance between having little or no external activation (the model reaches an equilibrium state that is largely independent of its initial state, which is not a cognitively realistic scenario), and having too much external activation (the spreading of activation adds very little new information, which again does not seem to be cognitively plausible).

**Figure 1:**
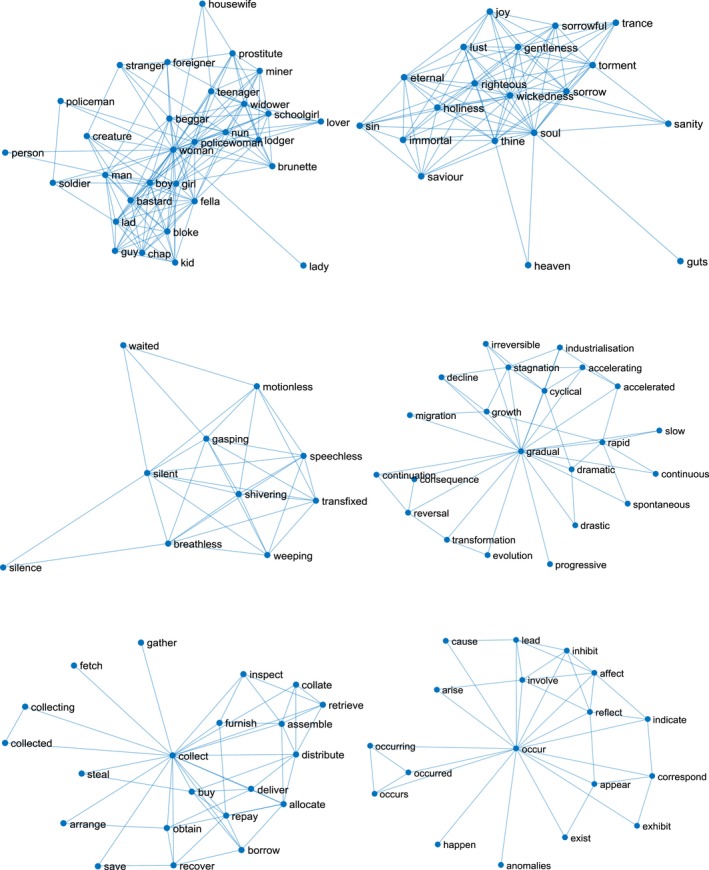
Local semantic neighborhoods for three concrete words (i.e., “woman”, “silent”, “collect”; on the left) and three abstract words (i.e., “soul”, “gradual”, “occur”; on the right), covered by the CBOW model. We include only very strong neighbors for each word (i.e., pairs of words with cosine similarity greater than 0.425).

**Figure 2:**
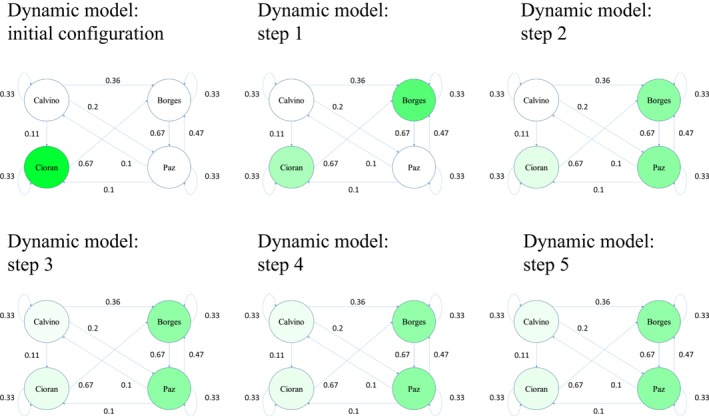
Toy example of the spreading of activation in our dynamic model. The network consists of four words and eight directional semantic associations between the words. The levels of activation are represented by the intensity of the colors for each word. Initially, only *Cioran* is activated; during step 1, *Borges* receives activation from *Cioran;* during step 2, *Paz* receives activation from *Borges*; during step 3, *Paz* and *Borges* exchange part of their activation, while *Cioran* and *Calvino* receive activation from *Paz*; during the remaining steps, the network reaches a state of equilibrium, such that the level of activation corresponding to each word remains constant.

We model the spreading of activation within the semantic network as occurring in discrete time steps, rather than being a continuous process, which allows us to express our models as a discrete‐time Markov chain, denoted as *MC*. In this way we can further assess whether the initial steps better capture tasks that only implicitly tap into semantic knowledge (such as the lexical decision task) whereas tasks that explicitly require semantic activation (such as semantic decisions, but also ratings of concreteness and imageability) correspond to later steps of the chain. The probability matrix underlying *MC* is represented by *DM* (i.e., the dynamic model), such that *DM* = (2 * *SM*_*NORM*_ + *I*_*N*_)/3. An important aspect to keep in mind is that, regardless of the chain's initial state, after a relatively small number of time steps, *MC* reaches a stable, fixed distribution, known as a steady‐state/equilibrium distribution. This means that in our analyses, we will focus only on the first few time steps in the evolution of the chain, given that the subsequent time steps provide little new information. An illustration of the structural and dynamic models is given in Figs. 1 and 2.

Let *S*_*k*_(*MC*) denote the state of *MC* at time step *k*. In most of our empirical validations, we are interested in the number of neighbors of word *w*_*i*_, at various distances from *w*_*i*_. More specifically, we partition *S*_*k*_(*MC*) into 10 deciles, such that each word *w*_*j*_ falls into one decile depending on its activation/probability, given by *S*_*k*_(*MC*). These quantiles, then, are the neighborhoods. The number of neighbors at each step *k* and in each decile *d,* denoted as *numNeigh*_*k,d*,_ forms the predictor for reaction times, response accuracies, and concreteness and imageability ratings. For modeling similarity judgments (i.e., “how similar/related are *w*_*i*_ and *w*_*j*_?”), we take the probability of *w*_*j*_ given the Markov chain that starts from *w*_*i*_, as well as the probability of *w*_*i*_ given the Markov chain that starts from *w*_*j*_. We look at both forward and backward probabilities because, whereas activation spreads in our network in an asymmetrical manner, we believe that similarity/relatedness judgements are largely symmetrical, although the issue of symmetry in (episodic and semantic) memory associations is still under debate (Kahana, 2002; Tversky, 1977).

The actual mathematical formulation of the structural and dynamic models, as well as of our measures of interest, involves going through the following steps:

1. Use the CBOW/GloVe/LSA model in order to obtain 300‐dimensional vector representations for all the words in a given set of size *N*, representations which we denote by *vecs*. The matrix *vecs* is of size *N *×* *300, such that each row corresponds to the vector associated with a given word.
2. Compute a similarity matrix *M*, of size *N*×*N*, from said vectors, using vector cosine as a measure of similarity between vectors, such that *M *= (*vecs*/||*vecs|*|) _*_ (*vecs*/||*vecs*||)^*T*^, where *T* denotes the matrix transpose, ||∙|| denotes the Euclidian norm (computed for each row), and/denotes element‐wise division.
3. Set to zero all the negative values in the cosine matrix, meaning that *SM*(*i,j*) = *M*(*i,j*), if *M*(*i,j*) > 0, and *SM*(*i,j*) = 0, otherwise.
4. Employ the matrix *SM* as a structural model.
5. Set to zero the diagonal elements of the matrix *SM*, then normalize its rows, such that each row sums to one. This means that *SM*_*NORM*_(*i,j*) = 0, if *i* = *j*, and *SM*_*NORM*_(*i,j*) = *SM*(*i,j*) / ∑_*k*_{*SM*(*i,k*) | 1 *≤*  *k  ≤  N* and *k ≠ i*}, otherwise.
6. Employ the matrix *DM* = (2 * *SM*_*NORM*_ + *I*_*N*_)/3 as the probability matrix for the Markov chain *MC* representing our dynamic model, where *I*_*N*_ is the identity matrix of size *N*.
7. Let *S*_*k*_(*MC*) denote the state of *MC* at step *k*. This state can be computed by raising DM to the power of *k*, meaning that *S*_*k*_(*MC*) = *DM* ^*k*^. Thus, for any row *i* and column *j*, the value *S*_*k*_(*MC*)(*i,j)* represents the probability that *MC* is in state *j*, at time step *k*, given that it started in state *i*. This probability gives us the amount of activation associated with word *w*_*j*_, at time *k*, following the initial presentation of word *w*_*i*_.
8. When modeling non‐relational tasks (e.g., lexical or semantic decision, imageability or concreteness rating), for any word *w*_*i*_ and time step *k* between 1 and 5, we compute *numNeigh*_*k,d*_(*i*) as the number of elements on row *i* of *S*_*k*_(*MC*) that have activations (i.e., probabilities) falling into the *d* ^th^ decile of all the activations in *S*_*k*_(*MC*). In other words, for *d = *1 and d = *10*, we count the weakest and the strongest neighbors of *w*_*i*_, respectively, while for any *d* between 2 and 9 we calculate how many of the neighbors have intermediate levels of activation. More formally, *numNeigh*_*k,d*_(*i*) is equal to the number of elements in the set {*S*_*k*_(*MC*)(*i,j*) | quantile(*S*_*k*_(*MC*), 10*(*d−*1)) < *S*_*k*_(*MC*)(*i,j*) *≤* quantile(*S*_*k*_(*MC*), 10*d)}, for 1 ≤  *j * ≤  *N*}, for 1 ≤  *k *≤* *5 and 1 ≤  *d *≤* *10. For consistency, we also perform an analogous count for the cosine similarity values in the matrix *SM*, resulting in a total of (5 + 1) * 10 = 60 predictors for each of the CBOW, GloVe, and LSA models.
9. When modeling relational tasks (e.g., similarity/relatedness rating), for any two words *w*_*i*_ and *w*_*j*_, and time step *k* between 1 and 5, we use the values *S*_*k*_(*DM*)(*i,j*) and *S*_*k*_(*DM*)(*j,i*) to represent the strength of the association between *w*_*i*_ and *w*_*j*,_, and that between *w*_*j*_ and *w*_*i*_, respectively. We obtain a total of 5 * 2 = 10 predictors for each of the CBOW, GloVe, and LSA models.

## 3 Model testing

### 3.1 Behavioral measures

We tested our models on a number of behavioral measures taken from existing sources. These are (a) lexical decision RTs and accuracy, for a subset of 2,328 words taken from Keuleers et al. (2012); (b) semantic decision RTs and accuracy for a subset of 2,639 words from Pexman et al. (2017) in which participants were asked to classify a word as either concrete or abstract; (c) concreteness ratings and (d) imageability ratings for the same words as (1) taken from Keuleers et al. (2012); (e) semantic similarity/relatedness ratings taken from Silberer and Lapata (2014; we selected 6,011/7,576 entries from SL), Bruni et al. (2014; we selected 2,835/3,000 entries from MEN), Gerz et al. (2016; we selected 3,326/3,500 entries from SimVerb‐3500), and Hill, Reichart, and Korhonen (2015; we selected 945/999 entries from SimLex‐999). For all these tasks, we selected all the words covered by our models and norms.

### 3.2 Baseline models

In order to assess the role of structural relationships among words and dynamic flow of activation, we first compared our models to a baseline model that included as many as possible of the other variables which are known to affect lexical and semantic decisions, or concreteness and imageability ratings. In order to evaluate our models conservatively, we crucially included a number of semantic and non‐semantic variables to assess whether our structural measures provide a fit above and beyond the other semantic predictors. The choice of the specific variables to include in the baseline model for each task is dictated by the availability of relevant norms as well as considerations regarding the specific task used. Then, we compared a combination of the baseline model, the ten neighborhood sizes from the structural models, and the ten neighborhood sizes from the individual steps of the dynamic models, with a combination of the baseline model and the structural models.

For the analysis of the lexical decision RT and accuracy, we used a baseline model including age of acquisition (Kuperman, Stadthagen‐Gonzalez, & Brysbaert, 2012), familiarity (Gilhooly & Logie, 1980; Stadthagen‐Gonzalez & Davis, 2006), log frequency, log contextual diversity (Van Heuven et al., 2014), semantic diversity (Hoffman et al., 2013), (squared) hedonic valence (Warriner, Kuperman, & Brysbaert, 2013), number of letters, Coltheart's N (i.e., the number of words that can be produced by substituting one letter of a given word, for any other, such that the result is a valid word; Coltheart, Davelaar, Jonasson, & Besner, 1977), orthographic Levenshtein distance (OLD20; the average orthographic editing distance between a word and its twenty closest neighbors in the lexicon; Yarkoni, Balota, & Yap, 2008), and phonological Levenshtein distance (PLD20; the average phonological distance between a word and its twenty closest neighbors in the lexicon; Suárez, Tan, Yap, & Goh, 2011). For the analysis of semantic decision RTs and accuracy, the baseline model included log frequency, semantic diversity, number of letters and orthographic Levenshtein distance, in order to attempt to replicate the findings by Pexman et al. (2017).

For the analysis of concreteness and imageability rating tasks, the baseline model included age of acquisition, familiarity, log frequency, log contextual diversity, semantic diversity, (squared) hedonic valence, number of letters, Coltheart's N, OLD20, and PLD20. Finally, for the analysis of semantic similarity/relatedness ratings, we omitted a baseline model, given that performance in these tasks has been shown to be very well captured by the information provided by distributional models alone (Baroni et al., 2014; Bruni et al., 2014; Pereira et al., 2016).

### 3.3 Results

For each behavioral measure, we assessed whether a purely structural model can fit the data better than a baseline model and then, crucially, whether further including spreading of activation (across five consecutive steps) provided any further improvement of the fit. In order to deal with the problem of multiple comparisons, we employed the Bonferroni correction when reporting the statistical significance of each result.

#### 3.3.1 Lexical decision

**Figure 3:**
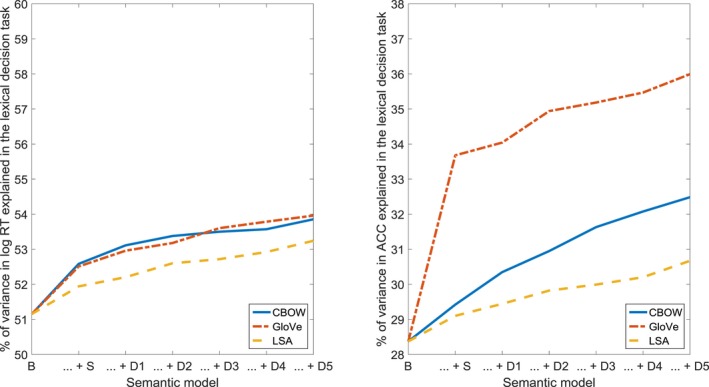
Percentage of variance in log response time (RT) and accuracy (ACC) in the lexical decision task, accounted for by the baseline model (B), the combination of the baseline model and the structural model (… + S), and the combination of the baseline model, the structural model, and consecutive steps of the dynamic model (… + D1 through … + D5).

The results for the lexical decision task are shown in Fig. 3 and Table 1. For log response time, the fit was improved by the addition of the structural models (CBOW, GloVe, and LSA), as well as by the inclusion of the first and second steps (CBOW), and of the third and fourth steps (GloVe), in the case of the dynamic models. For accuracy, a significantly better fit was obtained when adding the structural models (CBOW, GloVe), as well as the first step (CBOW), and steps two through five (CBOW, GloVe), of the dynamic models. These results suggest that the dynamics of the semantic network, as captured by our models, provide a complementary source of information regarding semantic processing in the lexical decision task.

**Table 1:** Results of model comparisons for predicting log response time and accuracy in the lexical decision task. The comparisons are between the combination of the baseline model and the structural model (B + S) versus the baseline model (B), as well as between the combination of the baseline model, the structural model, and individual steps of the dynamic model (B  +  S  +  D_1…5_) versus the combination of the baseline model and the structural model (B  +  S)

Simple Model	B	B + S	B + S	B + S	B + S	B + S
Enhanced model	B+S	B+S+D_1_	B+S+D_2_	B+S+D_3_	B+S+D_4_	B+S+D_5_
Degrees of freedom	10, 2307	10, 2297	10, 2297	10, 2297	10, 2297	10, 2297
*F* value (*p* value) for log RT						
CBOW	6.96 (< 0.0001)	2.59 (0.004)	2.87 (0.001)	1.40 (0.18)	1.97 (0.03)	2.11 (0.02)
GloVe	6.59 (<0.0001)	2.20 (0.02)	1.78 (0.06)	3.23 (0.0004)	2.98 (0.001)	2.30 (0.01)
LSA	3.79 (<0.0001)	1.26 (0.25)	1.62 (0.09)	1.45 (0.15)	0.76 (0.67)	1.03 (0.41)
*F* value (*p* value) for accuracy						
CBOW	3.42 (0.0002)	3.06 (0.0007)	3.21 (0.0004)	3.31 (0.0003)	4.43 (<0.0001)	4.44 (<0.0001)
GloVe	18.45 (<0.0001)	1.27 (0.24)	3.37 (0.0002)	2.95 (0.001)	2.94 (0.001)	3.16 (0.0005)
LSA	2.37 (0.01)	1.11 (0.35)	1.11 (0.35)	0.87 (0.56)	0.56 (0.85)	0.64 (0.78)

An additional interesting question is whether the models behave similarly for concrete and abstract words. In order to assess this, we divided our words into two classes, based on concreteness ratings, and ran separate analyses for each subset of words. Overall, it appears that the behavior of the models is largely comparable across the two word classes (see detailed results in the Appendix).

#### 3.3.2 Semantic decision

**Figure 4:**
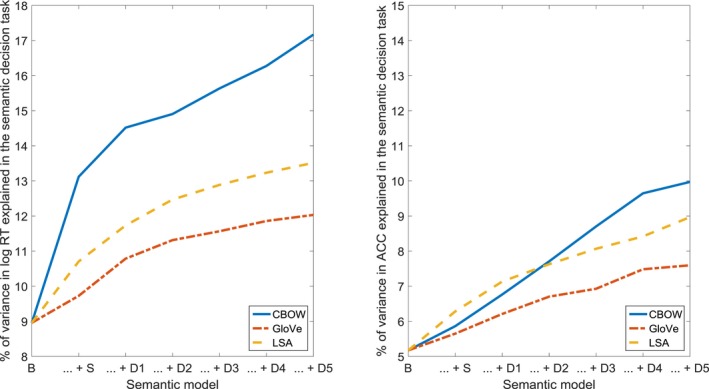
Percentage of variance in log response time (RT) and accuracy (ACC) in the semantic decision task, accounted for by the baseline model (B), the combination of the baseline model and the structural model (… + S), and the combination of the baseline model, the structural model, and consecutive steps of the dynamic model (… + D1 through … + D5).

The results for the semantic decision task are shown in Fig. 4 and Table 2. For log response time, the addition of the structural models significantly improved the fit in two out of three cases (CBOW, LSA). In the case of the dynamic models, the fit was ameliorated by the inclusion of step one (CBOW, GloVe, LSA), steps two and three (GloVe, LSA), and steps four and five (CBOW, GloVe). For accuracy, however, only the addition of one of the structural models (LSA), and of step one (CBOW, LSA) and step four (CBOW), improved the fit. It is important to note that our findings for log response time are in contradiction with the results of several previous studies (Pexman, Hargreaves, Siakaluk, Bodner, & Pope, 2008; Yap, Pexman, Wellsby, Hargreaves, & Huff, 2012; Yap, Tan, Pexman, & Hargreaves, 2011; Zdrazilova & Pexman, 2013), where no effects of neighborhood size and connectivity were detected. This discrepancy may come about because we perform a relatively fine‐grained analysis of neigborhood size, as a function of semantic distance, resulting in ten neighborhoods per word, while all the other studies only focus on (very) close neighborhoods, yielding one neighborhood per word. Also, we include both the structure and the dynamics of our semantic network, whereas the other approaches investigate only structural aspects.

**Table 2:** Results of model comparisons for predicting log response time and accuracy in the semantic decision task. The comparisons are between the combination of the baseline model and the structural model (B+S) versus the baseline model (B), as well as between the combination of the baseline model, the structural model, and individual steps of the dynamic model (B+S+D_1…5_) versus the combination of the baseline model and the structural model (B+S)

Simple Model	B	B+S	B+S	B+S	B+S	B+S
Enhanced model	B+S	B+S+D_1_	B+S+D_2_	B+S+D_3_	B+S+D_4_	B+S+D_5_
Degrees of freedom	10, 2624	10, 2614	10, 2614	10, 2614	10, 2614	10, 2614
*F* value (*p* value) for log RT						
CBOW	12.57 (<0.0001)	4.28 (<0.0001)	1.40 (0.18)	2.25 (0.01)	2.55 (0.005)	3.62 (<0.0001)
GloVe	2.24 (0.01)	3.11 (0.0006)	2.84 (0.002)	3.23 (0.0004)	3.07 (0.0007)	2.77 (0.002)
LSA	5.14 (<0.0001)	3.04 (0.0008)	2.66 (0.003)	2.58 (0.004)	1.97 (0.03)	2.34 (0.01)
*F* value (*p* value) for accuracy						
CBOW	1.92 (0.04)	2.53 (0.005)	2.36 (0.009)	1.79 (0.06)	2.49 (0.006)	1.52 (0.12)
GloVe	1.32 (0.21)	1.56 (0.11)	1.82 (0.05)	1.74 (0.07)	2.19 (0.02)	1.26 (0.25)
LSA	3.10 (0.0006)	2.39 (0.008)	2.16 (0.02)	1.78 (0.06)	1.74 (0.07)	2.20 (0.02)

#### 3.3.3 Concreteness and imageability ratings

**Figure 5:**
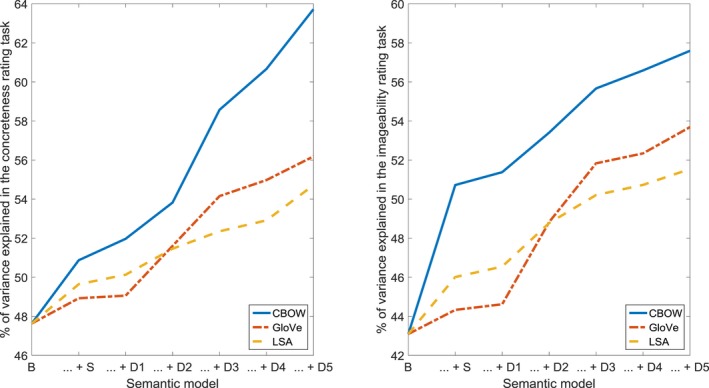
Percentage of variance in concreteness and imageability ratings, accounted for by the baseline model (B), the combination of the baseline model and the structural model (… + S), and the combination of the baseline model, the structural model, and consecutive steps of the dynamic model (… + D1 through … + D5).

For the concreteness and imageability ratings (see Fig. 5 and Table 3), the structural models (CBOW, GloVe, LSA), step one in the dynamic models (CBOW), and steps two through five (CBOW, GloVe, LSA), significantly improved the fit. Our findings clearly indicate that concreteness and imageability are reflected in both the structure and the dynamics of the semantic network.

**Table 3:** Results of model comparisons for predicting concreteness and imageability ratings. The comparisons are between the combination of the baseline model and the structural model (B+S) versus the baseline model (B), as well as between the combination of the baseline model, the structural model, and individual steps of the dynamic model (B+S+D_1…5_) versus the combination of the baseline model and the structural model (B+S)

Simple Model	B	B+S	B+S	B+S	B+S	B+S
Enhanced model	B+S	B+S+D_1_	B+S+D_2_	B+S+D_3_	B+S+D_4_	B+S+D_5_
Degrees of freedom	10, 2307	10, 2297	10, 2297	10, 2297	10, 2297	10, 2297
*F* value (*p* value) for concreteness						
CBOW	15.21 (<0.0001)	5.25 (<0.0001)	11.88 (<0.0001)	14.62 (<0.0001)	22.33 (<0.0001)	35.65 (<0.0001)
GloVe	5.84 (<0.0001)	0.61 (0.81)	7.64 (<0.0001)	17.75 (<0.0001)	20.61 (<0.0001)	23.08 (<0.0001)
LSA	9.21 (<0.0001)	2.26 (0.01)	3.02 (0.0009)	6.58 (<0.0001)	9.00 (<0.0001)	13.59 (<0.0001)
*F* value (*p* value) for imageability						
CBOW	35.71 (<0.0001)	3.11 (0.0006)	8.84 (<0.0001)	10.22 (<0.0001)	13.49 (<0.0001)	19.42 (<0.0001)
GloVe	5.11 (<0.0001)	1.21 (0.28)	12.28 (<0.0001)	26.25 (<0.0001)	26.69 (<0.0001)	33.10 (<0.0001)
LSA	12.46 (<0.0001)	2.30 (0.01)	4.97 (<0.0001)	8.03 (<0.0001)	15.42 (<0.0001)	17.13 (<0.0001)

#### 3.3.4 Semantic similarity/relatedness ratings

**Figure 6:**
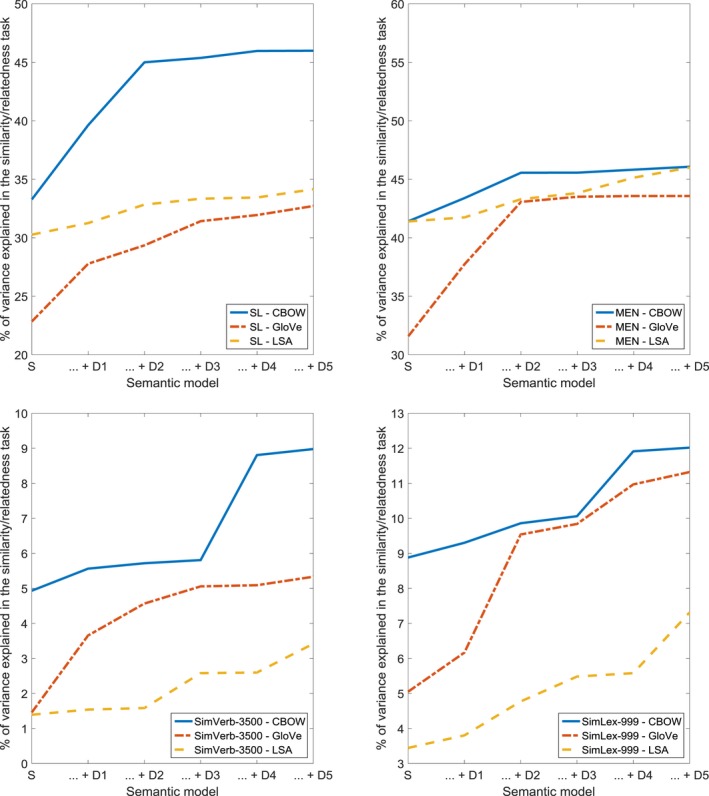
Percentage of variance in similarity and/or relatedness ratings (SL, MEN, SimVerb‐3500, and SimLex‐999), accounted for the structural model (S), and a combination of the structural model and consecutive steps of the dynamic model (… + D1 through … + D5).

For the semantic similarity/relatedness ratings (see Fig. 6 and Tables 4 and 5), the addition of any of the steps in the dynamic models (CBOW, GloVe, LSA) improved the fit to the SL dataset. For the MEN dataset, the fit was increased by the addition of step one (CBOW, GloVe, LSA), of steps two and three (CBOW, GloVe), as well as of steps four and five (CBOW, GloVe, LSA). Also, the addition of and of the steps in two of the dynamic models (CBOW, GloVe), ameliorated the fit to the SimVerb‐3500 dataset. In the case of the SimLex‐999 dataset, the inclusion of steps one and three (GloVe), as well as of steps four and five (GloVe, LSA) in the dynamic models significantly contributed to the model fit. These results seem to suggest that similarity/relatedness judgements correlate strongly with both the structure and dynamics of the semantic network underlying our models. Our findings hold across datasets covering a wide range of word frequencies, semantic relations, and parts of speech (but note the large difference in explained variance between SL/SEM and SimVerb/SimLex).

**Figure 7:**
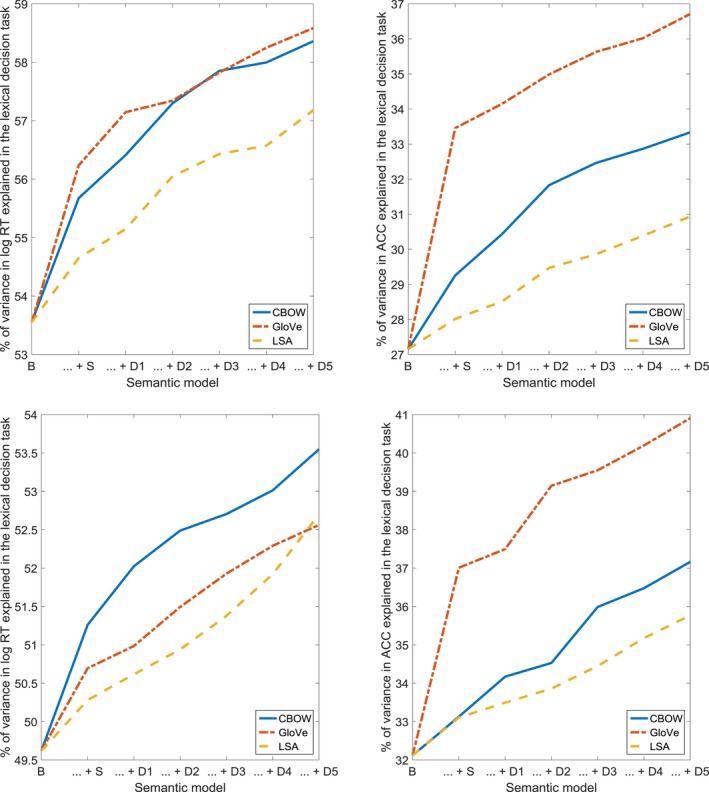
Percentage of variance in log response time (RT) and accuracy (ACC) in the lexical decision task, accounted for by the baseline model (B), the combination of the baseline model and the structural model (… + S), and the combination of the baseline model, the structural model, and consecutive steps of the dynamic model (… + D1 through … + D5). Top: results for concrete words only. Bottom: results for abstract words only.

**Table 4:** Results of model comparisons for predicting SL and MEN similarity/relatedness ratings. The comparisons are between the combination of the structural model and individual steps of the dynamic model (S+D_1…5_), and the structural model (S)

Simple Model	S	S	S	S	S
Enhanced model	S+D_1_	S+D_2_	S+D_3_	S+D_4_	S+D_5_
SL					
Degrees of freedom	2, 6007	2, 6007	2, 6007	2, 6007	2, 6007
*F* value (*p* value)					
CBOW	315.65 (<0.0001)	158.93 (<0.0001)	44.46 (<0.0001)	470.16 (<0.0001)	495.88 (<0.0001)
GloVe	205.17 (<0.0001)	130.59 (<0.0001)	8.94 (0.0001)	98.92 (<0.0001)	235.26 (<0.0001)
LSA	43.22 (<0.0001)	10.13 (<0.0001)	37.03 (<0.0001)	75.40 (<0.0001)	98.47 (<0.0001)
MEN					
Degrees of freedom	2, 2831	2, 2831	2, 2831	2, 2831	2, 2831
*F* value (*p* value)					
CBOW	49.66 (<0.0001)	36.19 (<0.0001)	7.93 (0.0004)	38.61 (<0.0001)	84.99 (<0.0001)
GloVe	140.14 (<0.0001)	47.80 (<0.0001)	70.31 (<0.0001)	267.00 (<0.0001)	290.32 (<0.0001)
LSA	8.48 (0.0002)	1.33 (0.26)	4.07 (0.02)	30.98 (<0.0001)	57.98 (<0.0001)

**Table 5:** Results of model comparisons for predicting SimVerb‐3500 and SimLex‐999 similarity/relatedness ratings. The comparisons are between the combination of the structural model and individual steps of the dynamic model (S+D_1…5_), and the structural model (S)

Simple Model	S	S	S	S	S
Enhanced model	S+D_1_	S+D_2_	S+D_3_	S+D_4_	S+D_5_
SimVerb‐3500					
Degrees of freedom	2, 3322	2, 3322	2, 3322	2, 3322	2, 3322
*F* value (*p* value)					
CBOW	11.08 (<0.0001)	12.12 (<0.0001)	13.17 (<0.0001)	13.43 (<0.0001)	6.00 (0.003)
GloVe	37.85 (<0.0001)	16.85 (<0.0001)	13.95 (<0.0001)	39.38 (<0.0001)	39.00 (<0.0001)
LSA	2.47 (0.08)	2.94 (0.05)	3.75 (0.02)	3.94 (0.02)	3.37 (0.03)
SimLex‐999					
Degrees of freedom	2, 941	2, 941	2, 941	2, 941	2, 941
*F* value (*p* value)					
CBOW	2.18 (0.11)	2.30 (0.10)	2.47 (0.09)	2.82 (0.06)	3.82 (0.02)
GloVe	5.60 (0.004)	1.04 (0.36)	13.09 (<0.0001)	19.54 (<0.0001)	15.38 (<0.0001)
LSA	1.75 (0.17)	1.81 (0.16)	3.73 (0.02)	7.70 (0.0005)	8.48 (0.0002)

**Table 6:** Results of model comparisons for predicting log response time and accuracy in the lexical decision task, for concrete words. The comparisons are between the combination of the baseline model and the structural model (B+S) versus the baseline model (B), as well as between the combination of the baseline model, the structural model, and individual steps of the dynamic model (B+S+D_1…5_) versus the combination of the baseline model and the structural model (B+S)

Simple Model	B	B+S	B+S	B+S	B+S	B+S
Enhanced model	B+S	B+S+D_1_	B+S+D_2_	B+S+D_3_	B+S+D_4_	B+S+D_5_
Degrees of freedom	10, 1142	10, 1132	10, 1132	10, 1132	10, 1132	10, 1132
*F* value (*p* value) for log RT						
CBOW	5.47 (<0.0001)	1.91 (0.04)	1.97 (0.03)	0.81 (0.62)	1.46 (0.15)	1.70 (0.08)
GloVe	7.00 (< 0.0001)	2.40 (0.008)	0.97 (0.47)	1.48 (0.14)	1.29 (0.23)	1.06 (0.39)
LSA	2.76 (0.002)	1.25 (0.25)	2.85 (0.002)	2.25 (0.01)	0.97 (0.47)	1.34 (0.20)
*F* value (*p* value) for accuracy						
CBOW	3.39 (0.0002)	1.92 (0.04)	2.57 (0.004)	2.06 (0.02)	2.54 (0.005)	2.41 (0.008)
GloVe	10.81 (< 0.0001)	1.19 (0.29)	1.88 (0.04)	1.55 (0.12)	0.77 (0.66)	1.25 (0.25)
LSA	1.36 (0.19)	0.79 (0.64)	1.63 (0.09)	1.28 (0.24)	1.33 (0.21)	1.14 (0.33)

**Table 7:** Results of model comparisons for predicting log response time and accuracy in the lexical decision task, for abstract words. The comparisons are between the combination of the baseline model and the structural model (B+S) versus the baseline model (B), as well as between the combination of the baseline model, the structural model, and individual steps of the dynamic model (B + S + D_1…5_) versus the combination of the baseline model and the structural model (B + S)

Simple Model	B	B+S	B+S	B+S	B+S	B+S
Enhanced model	B+S	B+S+D_1_	B+S+D_2_	B+S+D_3_	B+S+D_4_	B+S+D_5_
Degrees of freedom	10, 1144	10, 1134	10, 1134	10, 1134	10, 1134	10, 1134
*F* value (*p* value) for log RT						
CBOW	3.85 (<0.0001)	1.80 (0.06)	2.25 (0.01)	1.18 (0.30)	1.39 (0.18)	1.53 (0.12)
GloVe	2.50 (0.006)	0.67 (0.75)	1.04 (0.41)	1.41 (0.17)	1.41 (0.17)	0.99 (0.45)
LSA	1.52 (0.13)	0.77 (0.66)	0.50 (0.89)	1.11 (0.35)	1.12 (0.34)	1.04 (0.41)
*F* value (*p* value) for accuracy:						
CBOW	1.73 (0.07)	1.80 (0.06)	1.37 (0.19)	1.89 (0.04)	2.21 (0.02)	2.86 (0.002)
GloVe	8.88 (< 0.0001)	0.88 (0.55)	3.15 (0.0005)	3.06 (0.0008)	3.24 (0.0004)	3.07 (0.0007)
LSA	1.69 (0.08)	0.66 (0.76)	0.25 (0.99)	0.89 (0.54)	0.67 (0.75)	0.58 (0.83)

## 4 Discussion

We described here three models that take into account the structural properties of semantic networks, as well as their dynamic aspects, namely the flow of semantic activation generated by the automatic processing of individual words. By embedding both structure and dynamics, we could assess the effects of both direct and indirect (mediated) semantic relations between words, rather than limiting our analysis to strong, direct links. We found that our dynamic models predict results in all tasks we have considered above and beyond what is predicted by a model that takes into account not only a large number of lexical and sub‐lexical variables, but also semantic variables such as semantic diversity (Hoffman et al., 2013). Semantic diversity quantifies the similarity of the linguistic contexts in which a given word appears, has been found to account for a significant amount of variance in the lexical decision task (Hoffman & Woollams, 2015), and has been argued to capture important differences in semantic processing, especially between concrete and abstract words.

Of the three dynamic models, the ones based on CBOW and GloVe generated better results that the one based on LSA, in almost all the tasks (with the exception of the semantic decision task), in line with the finding that “word‐as‐context” models typically yield a higher performance than “document‐as‐context” models (Bruni et al., 2012; Riordan & Jones, 2011), and that “predict” models are usually superior to “count” models (Baroni et al., 2014; Pereira et al., 2016). Importantly, however, even for the poorest performing model, namely LSA, adding the spreading activation mechanism improved the model fit in all tasks (except for lexical decision, where the other models also did not fare very well). Thus, we have reason to believe that the advantages of considering the spread of activation are not tied to a particular type of distributional model. However, this does not mean that the choice of model is irrelevant: better structural models are likely to produce better dynamic models, given that the flow of semantic activation employs information encoded in the structure of the semantic network. In principle, the reverse is also conceivable: Better distributional semantics may increase the structural model's fit to human data to an extent that the dynamic model has no further contribution to make. However, our current results provide no reason to believe this to be the case.

We have shown that our models predict word processing in different tasks: both offline (untimed), semantic tasks such as providing ratings for concreteness and imageability, or for similarity/relatedness, but also online (timed) tasks that require more (semantic decision) or less (lexical decision) semantic information, both of which are assumed to recruit automatic spreading of activation across the semantic network (Dell, 1986; Roelofs, 1992). It is important to note here that although our models significantly predicted response time and accuracy in the lexical decision task, they are considerably more successful at predicting results from semantic rating and semantic decision tasks. A simple account for this difference is semantic decision and the other tasks tap into semantic processing to a greater extent than lexical decision. Importantly, however, the improvement in the fit of the models due to the dynamic steps was not limited to offline semantic tasks, but it was found also in online tasks (semantic decision and, to a lesser extent, lexical decision). This result indicates that the mechanism we have described here can be thought of in terms of automatic spreading of activation across the network.

Overall, our results show the usefulness and plausibility of joining distributional probabilistic modeling of semantics with dynamic processes. There are, however, limitations that we need to take into account. First, we make a number of simplifying assumptions in the models. For example, we assumed that all the words receive the same amount of initial activation; however, it is very likely that some words might benefit from a stronger initial activation than others, for a variety of reasons (e.g., due to increased imageability, valence, arousal, semantic and/or contextual diversity). We opted for this simplification because we simply do not know how much more activation particular words would receive. Another issue refers to the fact that, for the same reasons, we impose that the total amount of activation in our network remains constant, while it seems more cognitively realistic that activation first increases (i.e., semantic representations are accessed gradually), then reaches a plateau, and finally decreases (i.e., semantic representations are affected by competition for retrieval and time‐dependent decay, among other factors). Since modeling this type of dynamics requires the addition of several theoretical assumptions and model parameters, we do not tackle this issue here, for reasons of simplicity.

Finally, our dynamic models rely on a process of spreading activation in order to access higher‐order semantic relationships between words. Spreading of activation has long been considered as a psychologically plausible dynamic mechanism (e.g., Collins & Loftus, 1975; Dell, 1986). However, our implementation of spreading of activation is mathematically equivalent to a higher‐order transformation for examining the global structure of a word's neighborhood (rather than just the local structure used by the structural model). Thus, results similar to what we report here might be obtained through other computational means. For example, the graph structure of a word's local neighborhood is indicative of polysemy and homonymy (Panchenko, Simon, Riedl, & Biemann, 2016) so a direct analysis of neighborhood structure may yield predictions of human responses to the extent that these are affected by polysemy/homonymy. Future work could assess such alternative possibilities.

## 5 Similarities and differences with other models

Our dynamic models of semantic processing are similar to a number of other formal approaches to semantics, especially those put forward by Anderson (1983), De Deyne et al. (2016), and Steyvers et al. (2004). Moreover, there are a number of other approaches to semantic cognition which share our interest in exploring the role of weak and indirect semantic relations between words, and in analysing the dynamics of semantic processing. These approaches examine task performance in tasks such as intralist and extralist cued recall (Bruza, Kitto, Nelson, & McEvoy, 2009; Nelson, Kitto, Galea, McEvoy, & Bruza, 2013), discrete free association and synonym generation (Howard, Shankar, & Jagadisan, 2011), continuous free association (De Deyne & Storms, 2008a,b), verbal fluency (Hills, Jones, & Todd, 2012; Hills, Todd, & Jones, 2015), and lexical decision and similarity rating (De Deyne et al., 2013). Given the large methodological differences between these studies and ours, we do not discuss them here in more detail.

De Deyne et al. (2016) investigated, among others, some of the differences that exist between two popular types of semantic representations, namely those based on discrete and continued word association, and those based on word co‐occurrence in text corpora. The study also looked at the explanatory power of weak and/or indirect semantic relations, obtained using a spreading activation mechanism very similar to that employed by Anderson (1983). However, in contrast to our approach, the authors focused on the semantic categorization task and semantic similarity ratings, whereas we examine lexical and semantic decision, as well as concreteness, imageability and similarity/‐relatedness ratings. Another difference between their linguistic model and ours is the manner in which activation spreads: We assume that the global distributional overlap between a source word and a target word (i.e., their cosine similarity) determines the amount of activation transmitted, whereas De Deyne and his collaborators considered that this quantity is computed from the local probability of the source and target word directly co‐occurring in text (i.e., their pointwise mutual information). Also, in their dynamic model, the authors examined only the equilibrium state, as opposed to our approach, where we look at both the initial steps in the spreading of activation, and the activation profile corresponding to the equilibrium state.

Steyvers et al. (2004) examined the role of direct and mediated semantic associations in a number of episodic memory tasks, involving the evaluation of similarity between novel and studied items in a recognition‐based paradigm, the recollection of studied items in the extralist cued recall task, and the production of intrusions in the free recall task. Although the tasks rely primarily on episodic memory, the authors did not include any episodic component within their model, focusing instead on the semantic similarity between the words presented during the tasks. The associative structure of semantic memory was obtained from an extensive set of free association norms (Nelson, McEvoy, & Schreiber, 2004), which were first symmetrized, by combining cue‐target and target‐cue association probabilities, and then subjected to one of three treatments: (a) singular value decomposition for one‐step associations; (b) singular value decomposition for both one‐step and two‐step associations; (c) multidimensional scaling for associative chains involving one or more steps. By employing dimensionality reduction techniques and multi‐step associations, the resulting semantic network indexed both direct and indirect semantic relations between words, which is a defining feature of both their model and ours. Nevertheless, since our semantic representations are constructed automatically from large text corpora, we are not limited with respect to the number of words that we can include in our model, and we can make use of richer, more fine‐grained information than that which can be gleaned from free association norms, given that the latter usually collect only between 100 and 200 associations per normed word. Another difference between the models is that we look beyond one‐step and two‐step associations, by taking into account the effects of associative chains of lengths from one to five. Admittedly, Steyvers and his collaborators also explored the contribution of long associative chains, in the third version of their model, but they considered only the shortest chain between two words, whereas we employ all the chains between the same two words, regardless of length. A final difference is that we do not assume that semantic associations are symmetric (Tversky, 1977), especially given the strong asymmetry that is characteristic of free association probabilities (Nelson, Dyrdal, & Goodmon, 2005).

Anderson (1983) offered a unified account of various long‐term memory phenomena, with an emphasis on memory retrieval. Similar to our models, human memory was represented as a network of associations between meaningful units (e.g., words or sentences), such that the retrieval of task‐relevant units strongly depended on the spreading of activation (Collins & Loftus, 1975) between the elements of the network. However, there are at least two key differences between Anderson's model and ours. First, although Anderson mentioned that the spreading activation mechanism was inspired by research related to semantic priming, his model did not have a particular focus on semantic memory, given that the tasks to which the model were applied are mainly episodic. The author provided a detailed description of a number of aspects that are typically studied in the context of episodic memory, such as the occurrence of proactive and retroactive interference in the paired‐associate paradigm, the improvement of memory performance with practice, and the levels‐of‐processing effect. Moreover, the author indicated how to compute the strength of the associations formed between items that are presented in the same episodic context, but he did not offer a means of quantifying the semantic associations formed between items that are related in meaning. As a result, since our interest lies exclusively with semantic memory, many important aspects of Anderson's model (e.g., the nature and structure of the memory representations, as well as the encoding, maintenance, and forgetting mechanisms associated with them) are not present in our models. Secondly, the semantic associations between words are computed very differently between the models, since the quantities involved in computing the associations for the Anderson model depend on an a non‐relational variable (i.e., the “strength” of each word, based on the number and spacing of repetitions for that word), whereas the associations in our structural models are derived from a relational variable (i.e., the distributional similarity between pairs of words, based on the history of their co‐occurrence with other words).

Thus, overall, our dynamic models are similar to the three other models described above, in that they allow for indirect, mediated semantic relations between words to contribute to task performance, in a variety of semantic tasks. However, the models also differ significantly in a number of respects. First, given that most of the research on the dynamics of semantic activation has relied on free association norms (De Deyne et al., 2013; Nelson, McKinney, Gee, & Janczura, 1998), it is not surprising that two of the three related models used semantic representations derived from free association data. In contrast, our models operate with text‐based, distributional representations, which have the advantage of covering a considerably larger set of words, and of capturing a multitude of weak, but reliable semantic associations between words (De Deyne, Navarro, Perfors, & Storms, 2012), which are largely absent from free association norms. Also, since free association norms are task‐based, whereas text corpora are task‐independent, we believe that the semantic information accessed by our models is more general than that provided by free association norms. Second, the emphasis of our models is on the semantic process that extracts implicit information from the semantic representations, and on the additional data revealed at each step of the process. The related models did not examine the individual steps in the evolution of the semantic networks, but instead collapsed all the available information into a new, enhanced representation (e.g., in order to reduce the sparsity of the representations; De Deyne et al., 2016). Finally, in our approach, we look at the individual semantic neighborhoods associated with a large number of words, whereas the other approaches either investigated global neighborhoods (De Deyne et al., 2016) or were not directly concerned with network properties (Anderson, 1983; Steyvers et al., 2004).

The majority of the models presented here are based on distributional semantic models, and are in line with the mainstream approach of using co‐occurrences of words in text as the only data source from which to learn semantic representations and their neighborhood structure. It is the case, however, that a number of models have also been proposed that are not limited to linguistic information derived from texts, but also employ multimodal information, corresponding to sensory‐motor and emotional properties of words as data from which semantic representations are learnt (e.g., Andrews, Vigliocco, & Vinson, 2009; Bruni et al., 2014). These grounded (or embodied) models have been shown to provide better fit to behavioral data than models based solely on linguistic data. For example, Andrews et al. (2009) found that a Topic model (see Griffiths, Steyvers, & Tenenbaum, 2007) trained on both text and speaker‐generated features (covering perceptual, motor and affective properties of referents) was better at predicting semantic effects in speech error data (specifically semantic errors among slips of the tongue), as well as in semantic priming experiments and in word association norms. One might wonder therefore if the structure of the neighborhoods and the effect of spreading activation would be different in models of this type. We leave this question for future studies.

## 6. Conclusions

We have shown here that by supplementing state‐of‐the‐art text‐based models of semantic structure with relatively standard processing assumptions, these models can provide a much better fit to behavioral data from word processing tasks that require the use of semantic information (ratings of concreteness/imageability, semantic similarity/relatedness, semantic decision), but also for tasks such as lexical decision, for which semantic information plays a secondary role. The improvement from structural models alone is especially important given the large number of lexical and semantic variables we had already included in most of our baseline comparison models. Thus, our work demonstrates that by bringing together large scale probabilistic models of semantic representations and processing models we can better account for a variety of behavioral results. Moreover, the distributional models we chose cover a representative selection of some of the most frequently used model architectures (e.g., “count” vs. “predict”; Baroni et al., 2014; “word‐as‐context” vs. “document‐as‐context”; Riordan & Jones, 2011), suggesting that the gains of adding processing assumptions are not tied to a particular model or task. Our results extend those obtained by De Deyne et al. (2016), who used a similar methodology, but focused only on one type of text‐based model and two semantic tasks.

An important implication resulting from our findings is that dynamics are important and useful when modeling semantic behavior. As a result, network analyses of semantics can be easily improved by combining structural and processing assumptions, either in a direct manner (e.g., via spreading activation, in neural network models, or multi‐step inference, in probabilistic models), or in an indirect way (e.g., by examining shortest path, flow and random process‐based centrality measures; De Deyne et al., 2016; Griffiths, Steyvers, & Firl, 2007; Steyvers et al., 2004; for a technical introduction, see Koschützki et al., 2005).

## Appendix 1

### 1.1

To investigate whether our structural and dynamic models might have a different impact for concrete and abstract words, we performed a median split on concreteness for the 2,328 words from the lexical decision task, and tested our models separately on the subset of concrete words and that of abstract ones. The results for the lexical decision task are shown in Fig. 7 and Tables 6, 7. For log response time, the addition of the structural model improved the fit of the regression model for the concrete words (CBOW, GloVe, and LSA), as well as for the abstract words (CBOW, GloVe). In the case of the dynamic model, concrete words only benefited from the inclusion of the first step (GloVe) and the second step (LSA). In contrast, for accuracy, the fit was significantly increased by the addition of the structural model for concrete words (CBOW, GloVe), and for abstract words (GloVe). For the dynamic model, the fit was improved by the inclusion of steps one, four, and five (CBOW), in the case of the concrete words, and of steps two through four (CBOW, GloVe), as well as that of step five (CBOW), in the case of abstract words.

The results seem to suggest that the structural models are more predictive of task performance for concrete words than for abstract words, which the opposite pattern holds in the case of the dynamic models. One potential explanation for this finding is that understanding abstract words requires deeper, more elaborate processing than for concrete words.
